# “Plis de passage” Deserve a Role in Models of the Cortical Folding Process

**DOI:** 10.1007/s10548-019-00734-8

**Published:** 2019-10-03

**Authors:** Jean-François Mangin, Yann Le Guen, Nicole Labra, Antoine Grigis, Vincent Frouin, Miguel Guevara, Clara Fischer, Denis Rivière, William D. Hopkins, Jean Régis, Zhong Yi Sun

**Affiliations:** 1 Neurospin, CEA, Paris-Saclay University, 91191 Gif-sur-Yvette, France; 2 MD Anderson Cancer Center, University of Texas, 1515 Holcombe Blvd., Houston, TX 77030 USA; 3 INS, CHU La Timone, Aix-Marseille University, 264, rue Saint Pierre, 13385 Marseille, France

**Keywords:** Cortical folding, Sulcal roots, Sulcal pits, Pli de passage, Central sulcus

## Abstract

Cortical folding is a hallmark of brain topography whose variability across individuals remains a puzzle. In this paper, we call for an effort to improve our understanding of the pli de passage phenomenon, namely annectant gyri buried in the depth of the main sulci. We suggest that plis de passage could become an interesting benchmark for models of the cortical folding process. As an illustration, we speculate on the link between modern biological models of cortical folding and the development of the Pli de Passage Frontal Moyen (PPFM) in the middle of the central sulcus. For this purpose, we have detected nine interrupted central sulci in the Human Connectome Project dataset, which are used to explore the organization of the hand sensorimotor areas in this rare configuration of the PPFM.

## Plis de passage

**Fig. 1:**
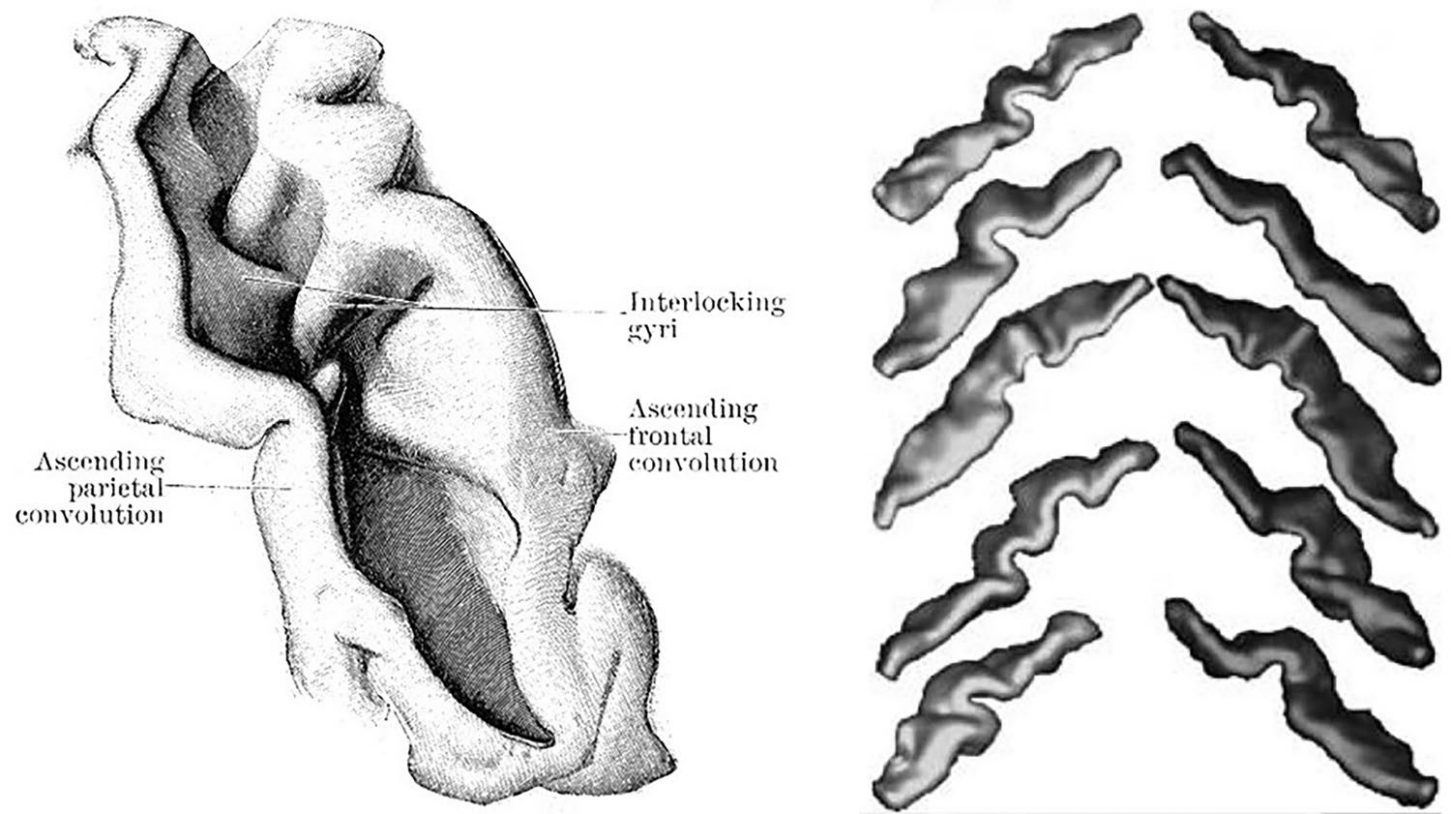
Left: drawing of the gyri buried in the walls of a central sulcus which become apparent when the lips of the sulcus are drawn apart (Cunningham 1903). Right: negative prints of five pairs of central sulci visualized from inside the brain and showing different patterns of buried gyri, courtesy of Mangin et al. 2004

**Fig. 2:**
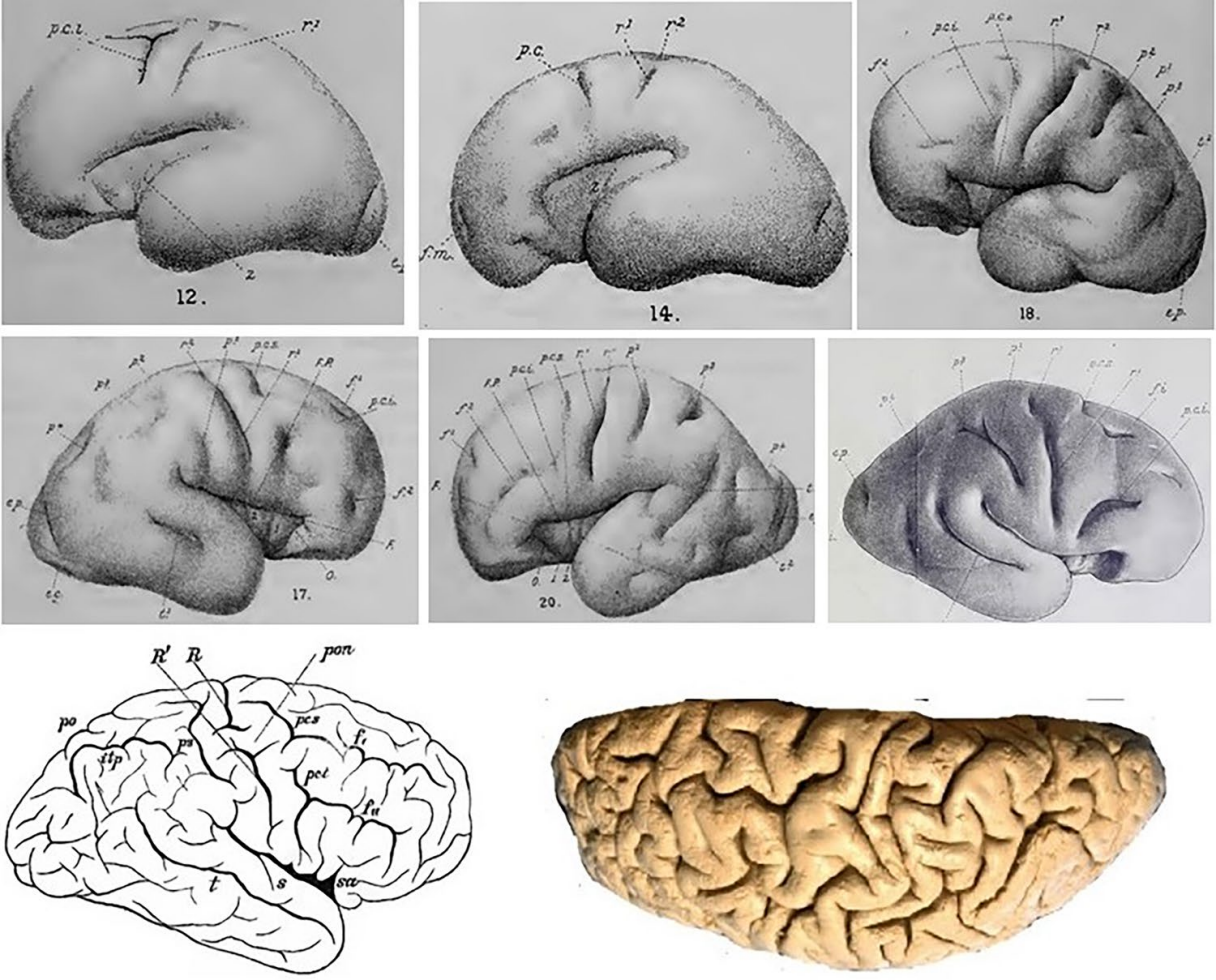
Top: 6 of the 39 hemispheres collected by D. J. Cunningham between the 5th and 7th months of development, showing the apparition of the central sulcus from two separated folding seeds (Cunningham 1890a, 1897). The lower portion (R1) appears first in the form of a shallow oblique groove which represents the two-thirds of the fully formed sulcus. The upper portion (R2) makes its appearance in the form of a deep pit between the upper end of the lower portion and the margin of the hemisphere. A faint furrow often runs over the summit of the elevation separating the two primitive portions. This double seed growing pattern is however not systematic. Bottom: Most of the time, as development goes on, this double seed pattern gets buried in the depth of the fissure, but in less than 1% of the subjects, the two portions do not merge, as in these 2 examples found in the literature, courtesy of Wagner 1864; Sernoff 1887; Schweizer et al. 2014

Cortical folding is a hallmark of brain topography whose variability across individuals remains a puzzle. We no longer compare cortical folding patterns to the coils of the small intestine, but deciphering the morphology of a given brain with the standard nomenclature of sulci and gyri is often full of obstacles. Long primary sulci can be split into pieces that can be recombined in nonstandard ways to create unusual folding patterns, which do not show a clear match with this nomenclature (Ono et al. 1990; Regis et al. 2005). In the middle of the nineteenth century, the anatomists inferring the first road maps of the sulci and gyri of the human brain introduced a concept helping to overcome the difficulties resulting from these interruptions: they described that some interconnecting gyri usually buried in the depth of the main furrows (see Figs. 1, 2) can emerge at the surface of the brain in some individuals. The French anatomist Gratiolet coined first the terms “pli de passage” (Gratiolet 1854; Parent 2014). Later on, the buried gyri were called annectant, bridging or transition convolutions in the English literature, and also submerged gyrus, or submerged gyral passage in recent publications (Germann et al. 2005; Zlatkina and Petrides 2010; Huntgeburth and Petrides 2012; Segal and Petrides 2012; Sprung-Much and Petrides 2018). While these plis de passage seem to appear during the earliest stages of development, which shall lead to regard them as candidates for being a distinctive character of the cortex organization, they did not receive a lot of attention. In this paper, we call for an effort to improve our understanding of the pli de passage phenomenon, and suggest that it could become an interesting benchmark for models of the cortical folding process.

**Fig. 3:**
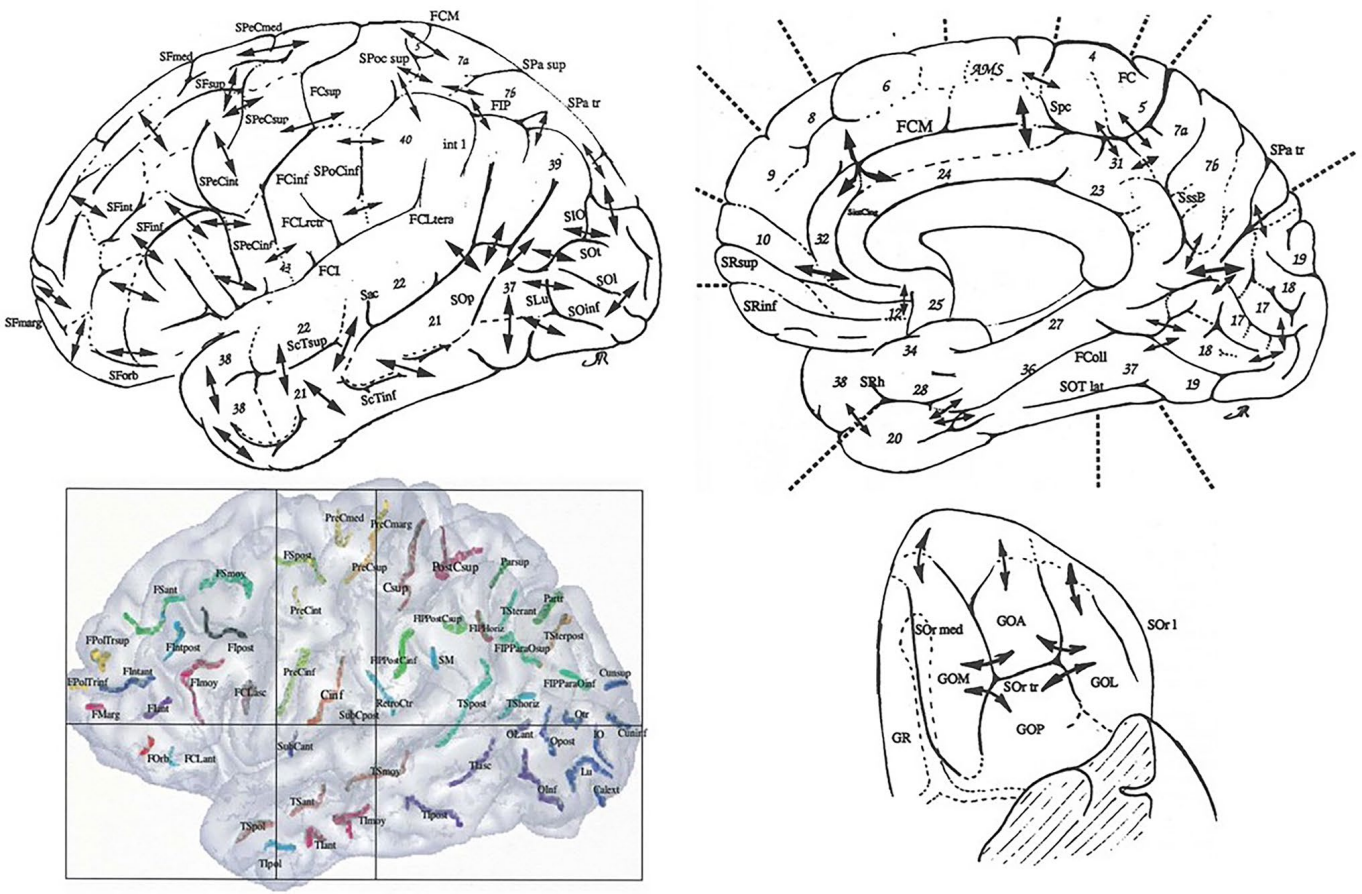
Top and lower right: Map of the plis de passage denoted by double arrows (aggregation of drawing of Jean Régis (1994)), Lower left: Map of the sulcal roots separated by superficial or buried gyri, courtesy of (Regis et al. 2005). Names are abbreviations of a French nomenclature of sulci and sulcal roots. Numbers refer to Brodmann areas

To our knowledge, the first comprehensive effort to map the plis de passage throughout the whole human cortex was performed by Jean Régis, a French neurosurgeon, during his MD thesis (Régis 1994) (cf Fig. 3). Each pli de passage reported in his map was inferred from the observation of a frequent interruption of the folding pattern in a specific spatial localization, often consistent with the disconnected initial folding patterns observed during development (Regis et al. 1997, 2005). In fact, the more usual mode of development of the main sulci is not in the form of a long continuous groove in the bottom of which plis de passage make their appearance latter on, but in the form of distinct and isolated pieces (Cunningham 1892) (see Fig. 2). The bridges of cortex which intervene between the isolated portions of a developing sulcus are ultimately buried down as the pieces run into each other; but they are not, as a rule, completely obliterated. In the bottom of the fissures they still show as deep annectant gyri (see Fig. 1).

While the map of the plis de passage is a very useful guideline when trying to decipher the idiosyncrasies of individual brains, the design of algorithmic strategies to manipulate explicitly the cortical folding patterns required to flip the concept, in order to deal with folds rather than gyri. In fact, the cortical gyri are difficult to define explicitly because of the lack of elementary building blocks: the gyri are in continuity with each other, which prevents to give them a straightforward geometrical definition fitting any configuration, especially with regard to the interruptions of the main furrows.

## Sulcal Roots and Sulcal Pits

Hence, in the nineties, computational anatomy focused on the cortical sulci, especially the main furrows, which are made up of aggregations of elementary folds with simple geometric definitions (Mangin et al. 2015a, b). Note however recent attempts at defining gyrus-based building blocks of the folding patterns (Chen et al. 2017; Duan et al. 2017). Modeling the cortical sulci and their interruptions led to define a new map based on the concept of sulcal roots (Régis 1994; Regis et al. 1997, 2005) (cf Fig. 3). Conceptually, the sulcal roots are entities supposed to correspond to seeds of the folding process disseminated across the cortical surface. The sulcal root dual map was inferred from the map of the plis de passage from a virtual configuration where each pli de passage is reaching the surface of the brain, creating a local interruption of the folding pattern. Then the sulcal roots can be represented by the connected components of the folding pattern in this virtual map endowed with the maximum number of interruptions. Some of the sulcal roots can be observed explicitly in utero at the first stage of the folding, but this is far to be systematic.

The concept of sulcal roots is very powerful for designing generative “grammatical” models, which can father any folding pattern configuration. In a way, these sulcal roots correspond to some kind of letters supposed to appear only one time in each brain, and the main sulci correspond to words made up from these letters and corresponding to the most frequent connections observed in the general population. Then, in the single brain template framework proposed by most anatomy books, each sulcal root is used in only one word of the nomenclature. But dealing comfortably with unusual folding patterns may require an extended vocabulary (Mangin et al. 2016). Several very interesting proposals of extended glossary based on regional folding patterns have been proposed (Steinmetz et al. 1990; Germann et al. 2005; Zlatkina and Petrides 2010; Huntgeburth and Petrides 2012; Segal and Petrides 2012; Plaze et al. 2015; Sprung-Much and Petrides 2018; Snyder et al. 2019): each reported pattern often corresponds to a specific configuration of the regional plis de passage, leading to alternative connectivity of the regional sulcal roots. This pattern glossary can be used to perform a subdivision of the population relative to the geometry of the cortical morphology. The hope is that such a subdivision provides a proxy to architectural differences. In the future, unsupervised clustering may contribute to the inference of a comprehensive dictionary of the folding patterns (Sun et al. 2007, 2009; Duan et al. 2019).

Because of the lack of data about the early stages of the normal folding dynamics, however, testing whether sulcal roots are reproducible and actually correspond to a key element of the folding process is difficult. Highly premature birth occurring at the early stage of the folding process is a very attractive source of data, but cannot be considered as normal development (Dubois et al. 2008; de Vareilles et al. 2019). The development of in utero imaging could rapidly change the situation (Lefèvre et al. 2015; Makropoulos et al. 2018). Furthermore, longitudinal acquisitions could lead to explore further the idea that the folding dynamics embed some kind of seeds with higher folding speed (Lefèvre et al. 2009; Xia et al. 2019).

Nevertheless, sulcal roots are mainly a concept used to decompose the folding dynamics. Hence sulcal root maps are difficult to match with the actual cortical folding patterns. Therefore, the community has moved to the notion of sulcal pits, the locally deepest points of the cortical surface, also corresponding to the local depth maxima along the bottom lines of the sulci (Lohmann et al. 2008; Im et al. 2010; Auzias et al. 2015). Intuitively, these deepest points could correspond to the places where the folding process begins and be intimately associated with the concept of sulcal roots. This is however also very difficult to test. Anyway the exact location of the roots in the folding pattern is not a crucial issue. The key contribution of the sulcal root/pit idea is the possibility to decompose the global sulcal pattern into reproducible building blocks overcoming the interruptions of the main sulci. An alternative but similar approach consists in decomposing the cortical surface into basins inspired by the watershed geographical notion (Lohmann and von Cramon 2000; Kruggel 2018). Sulcal pit maps have been inferred from large populations (Lohmann et al. 2008; Im et al. 2010; Auzias et al. 2015) including infants (Meng et al. 2014; Im and Grant 2019), highlighting strong similarities with the sulcal root maps and a striking reproducibility across populations and ages. It is now possible to quantify sulcal pits heritability, which ranges between 0.2 and 0.5 (Le Guen et al. 2018a). In a near future, sulcal pits configurations could be related to genetic variants providing insight into neurodevelopmental disorders (Ortinau et al. 2018; Im and Grant 2019).

## Mechanical and biological folding models

Why the cerebral cortex folds has fascinated and mystified some neurobiologists for a long time (Cunningham 1890c; Welker 1988; Van Essen 1997; Striedter et al. 2015). A large variety of intuitive generative mechanisms have been proposed but most of them have been dismissed during the last decades to give rise to a modern twofold vision of the phenomenon (Llinares-Benadero and Borrell 2019). The first point of view is purely mechanical, modeling the old intuition that the outside layer of the cortex increases by surface extension at a pace that cannot be compensated without folding by the slower volumetric extension of the inner part (Jelgersma 1889; Cunningham 1890c; Richman et al. 1975; Toro and Burnod 2005). Recent theoretical developments along that line have shown that realistic folding geometries can be obtained from simulations of the mechanical instability driven by the tangential expansion of the grey matter of the cortical mantle (Tallinen et al. 2014, 2016). Further theoretical developments aim at refining the mechanical modeling taking into account for instance the stiffness and elasticity of the different tissues (Kroenke and Bayly 2018; da Costa Campos et al. 2019).

An interesting question is whether a simplistic mechanical process is sufficient to generate some global features like the principal directions of the folds, which share striking similarities with meridian and parallel on earth (Régis 1994; Toro and Burnod 2003; Regis et al. 2005; Clouchoux et al. 2010; Auzias et al. 2013), or the folding wavelength that can be observed for instance in the periodicity of the sulcal pits in the meridian and parallel directions. The folding wavelength is clearly related to cortical thickness, which explains some of the cross species variability (Toro and Burnod 2005; Mota and Herculano-Houzel 2015; Llinares-Benadero and Borrell 2019). An extreme mechanical point of view leads to consider that the folding pattern could be mainly related to the initial geometry of the cortex (Bohi et al. 2019) and a few parameters like cortical thickness, the differential of expansion between grey and white matter, and mechanical properties. Hence it is sometimes speculated that the meridian/parallel organization could simply result from the directions of the two principal curvatures of the smooth cortical surface (Tallinen et al. 2016). It is even proposed that “mechanical morphogenesis”, namely the capacity of homogeneously growing elastic tissue to produce complex shapes, could play an important role in the segregation of the neocortex through the exploitation of the modules defined by the folding pattern (Foubet et al. 2018; Heuer and Toro 2019).

While the modern mechanical models represent an important step forward, none of them can generate the stereotyped folding pattern of the human cortices featured for instance in the sulcal pit map. It is difficult to believe that this will happen as long as the mechanical models rely on parameters spatially homogeneous throughout the cortical surface. Spatial and temporal heterogeneities, for instance in the expansion differential between the outer layer and the rest of the cortex, seem mandatory to select a specific reproducible pattern from the infinite number of attractors of the folding dynamics. Hence, a key issue is the biological substrates of the expansion differential (Borrell 2018; Kroenke and Bayly 2018). This is still a relatively open issue, but major advances have occurred during the last decade (Llinares-Benadero and Borrell 2019). The rapid tangential growth of the outer layer is probably strongly related to the differentiation of the neurons of the cortical plate, the growth of their cell bodies and of the surrounding neuropil (formation of axons, dendrites and synapses) (Wang et al. 2017). Hence, the microstructural differences across architectonic areas and their various maturation timelines probably father some heterogeneities in the maps of expansion differential across the cortical surface.

But before all, It seems that some variations in the initial packing of the neurons in the cortical plate existing after the migration from the subventricular zone gradually disappear during the tangential expansion leading to folding. Provided that these packing heterogeneities are similar across individuals, the higher expansion rate of the high neuron density areas may explain the existence of systematic seeds of folding in the spirit of the sulcal root concept. It has been shown that the variations in the packing of neurons originate from neurogenesis and neuron migration and are specific to folded mammal species (Kriegstein et al. 2006; Reillo et al. 2011; Borrell and Calegari 2014). Some regions of the subventricular zone enjoy higher neurogenesis rates, which result into greater surface expansion after the neuron migration into the cortical plate. Note however that the mechanism underlying the surface expansion may be more complex than what is embedded in the current mechanical models: progenitor cells located in intermediate zones of the subplate could lead to a tangential dispersion of the neurons occurring already during their radial migration rather than only from the cortical plate expansion (Llinares-Benadero and Borrell 2019). To conclude, it seems that the chronology of activity of the different sulcal roots may be strongly related to the chronology of neurogenesis.

A “protomap” of the primary folding pattern seems to exist at the level of the subventricular zone, before the neuronal migrations leading to the construction of the cortical mantle take place (de Juan Romero et al. 2015). This protomap, which is assumed to result from heterogeneous gene expressions, could be the primal sketch of both the differentiation of the cortex into architectonic areas and the primary folding pattern (Kostovic and Rakic 1990; Sur and Rubenstein 2005; Reillo et al. 2011). It would be really fascinating to be able to test whether the grid of sulcal pits/roots of the human brain is related to alternating blocks of high and low genetic expression patterns in the meridian and parallel directions (Lui et al. 2011; Albert and Huttner 2015; de Juan Romero et al. 2015). Since protogyral areas of the protomap, which will become gyri, correspond to an increased multiplication of neurons, they may contain the expansion maxima responsible for selecting the primary attractor in the folding dynamics. Therefore, by combining the mechanical model derived by the physicists with the protomap model from biology, simulations could resemble actual brains.

## Back to the plis de passage

Sulcal pits, sulcal basins and sulcal roots correspond to a wide variety of local folding geometries: a focal pit, an elongated groove, a T-shape configuration, or a more complex cushion button configuration. This variability in appearance is probably resulting from the variety of geometrical configurations of the seeds of higher tangential expansion surrounding them. Our building blocks of the folding patterns look like pieces of the boundaries of a parcellation of the cortex, and not like elementary architectural modules coded in the protomap or emerging from cortical maturation. Therefore it is tempting to flip back the point of view from folds to gyri, to try to pinpoint building blocks of the cortical morphology that would be easier to relate to the modern biological models of the folding process. Plis de passage are very good candidates even if they account only for a small part of the whole gyral morphology: they have a very focal localization, a clear geometry and they present a striking variability across individuals that has to be explained by the models. Each pli de passage may be related to one elementary feature of the protomap or of the cortical architecture.

Very few plis de passage have been studied in the literature. Therefore, in the following, we will focus on the PPFM, the “pli de passage frontal moyen”, described initially by Broca as a bulge into the middle of the central sulcus (Broca and Pozzi 1888). Later on, Cunningham spoke about a shallowing of the fissure and a deep interlocking of its adjacent walls (see Fig. 1) (Cunningham 1892; White et al. 1997). According to him, two of the interdigitating gyri are supposed to be always larger and more pronounced than the others, and in a considerable number of cases they unite at the bottom of the sulcus in the form of a distinct deep gyrus, which constitutes a marked interruption in its floor. All gradations between a mere shallowing with an interlocking of the adjacent walls of the fissure and the presence of a distinct deep annectant gyrus are met with.

The PPFM has become a subject of interest with the advent of functional imaging, because several studies have shown striking relationships with the hand sensorimotor areas (Yousry et al. 1997; Sastre-Janer et al. 1998; Boling et al. 1999; Boling and Olivier 2004). Note that the PPFM gathers two entities, a gyrus located in the precentral (frontal) wall of the central sulcus, supposed to be mainly associated to the motor hand area (Boling et al. 1999), and a gyrus located in the postcentral (parietal) wall of the central sulcus, supposed to be mainly associated to the sensory hand area (Boling and Olivier 2004). The neuroimaging community frequently uses the terms “hand knob” to denote the omega-shaped knob observed in the central sulcus in MRI axial sections and corresponding to the motor precentral part of the PPFM (Yousry et al. 1997). In some subjects, the knob has the shape of an epsilon in the MRI axial sections, which may correspond to configurations where it is shaped by two buried gyri located in the precentral wall of the sulcus. To our knowledge, the exact localization of the hand motor area for epsilon configurations has not been described yet.

Knowing now that the buried PPFM has a clear meaning relative to functional architecture, let us go back to its formation according to Cunningham (see Fig. 2) (Cunningham 1890c, 1897). Note first that Cunningham’s description of the development of the central sulcus is inferred from the observation of 39 hemispheres of his collection. Hence his “developmental movie” is purely virtual, based on some kind of mental interpolation, and can be questioned. In many cases, central sulcus arises in the form of a continuous groove, which is steadily deepen by the upheaval of its bounding banks. But frequently, it takes origin in two portions. Of these the lower and longer part makes its appearance first as a continuous groove. The upper part has an independent origin. It first shows as a slight depression, which widens and deepens and is separated from the lower part of the furrow by a high bridge of cortex. A faint groove then runs over the surface of this bridge and connects the two parts. In course of time the bridge disappears from the surface as the connecting groove cuts deeper and deeper into it, but in the after-growth of the brain it is retained in the form of an annectant gyrus. In very rare cases the two original portions of the central sulcus remain distinct throughout life, and the intervening bridge of cortex remains on the surface. Heschl, who examined 2174 hemispheres, found the interrupted form of the central sulcus only six times (Heschl 1877). Eberstaller met with it twice in 200 brains (Eberstaller 1890). A few other cases were reported in the literature (Schweizer et al. 2014), but the general impact seems around only 0.5% of the hemispheres.

Let us now try to interpret the development of the PPFM in the light of the modern biological models of cortical folding. This interpretation will be pure speculation, but the goal is to highlight the kind of hypotheses that could be put forward when trying to model the pli de passage development. The first event of the chain is the birth of the lower sulcal root of the central sulcus, a priori resulting from the tangential expansion of at least one of the two protogyri, which will sculpt the sulcus (precentral and postcentral), and a priori mainly in the direction orthogonal to the sulcus. According to Cunningham, the postcentral gyrus shows a “greater growing energy” than the precentral gyrus, which results in a tilt of the central sulcus orientation (Cunningham 1890b). Then, in some subjects, the lower sulcal root elongation toward the top of the brain is stopped by a piece of cortex bridging between the precentral and the postcentral gyri. This bridge seems endowed itself with a tangential expansion, maybe perpendicular to the first one, that can compete with the expansion generating the lower root. Later on, the folding action of this first expansion phenomenon, which seems to follow a chronological gradient from the bottom to the top of the brain, reappears at the level of the upper root. Most of the time, this initial expansion finally becomes faster than the second one leading to a fusion of the two sulcal roots.

The speculation above raises several questions about the origin of the expansion phenomena. The expansion of the precentral and of the postcentral gyri seem to be a primary phenomenon, which is a good candidate for being encoded in the genetic protomap. The very same pattern is observed in all primates (Heuer et al. 2019) and corresponds to the building of the primary motor and somatosensory systems. It is less clear why the hand areas would grow earlier or faster than the surrounding areas. It could be related to heterogeneities in the developmental trajectories of the sensorimotor subsystems across the body, the hand being an early bird. Cunningham himself suggested that the cortex must grow in accordance with the functional activity that is displayed in its different parts, leading to heterogeneities of the surface extension (Cunningham, 1890b, c). Hence, one may assume that the neuropil of the hand areas is developing earlier or faster, leading to the apparition of the bridging gyrus. Note that in a population born with a missing hand, it was observed that the contralateral central sulcus was often flat, without clearly demarcated hand knob (Sun et al. 2017).

An alternative hypothesis is the existence of a hand-dedicated neuron proliferation area in the protomap. Note that whenever the meridian/parallel organization of the sulcal roots corresponds to a grid-like 2D pattern in the protomap, it would make sense to have parallel axes of neuron proliferation orthogonal to the meridian axes corresponding to the precentral and postcentral gyri. Cunningham stressed the fact that the central sulcus development must be related to the development of the other two meridian sulci, the precentral and the postcentral sulci, which appear in two distinct pieces (Cunningham 1890b). It seems that an axis of neuron proliferation may cross in the middle of the three meridian sulci. Note also that the “hand knob” is clearly shaped in great apes but not in old world monkeys, which may rely on a genetical origin (Hopkins et al. 2014). Finally, when describing the central sulcus, Broca mentions three plis de passage (Broca and Pozzi 1888) (cf Fig. 3): the PPFM in the middle and two other plis above and under the sulcus, which may belong to two other axes in the same parallel direction. A grid-like protomap based on a simple 2D morphogenesis system may be underlying the folding process.

## Interrupted Central Sulci

It is interesting to extrapolate the above speculations to the rare cases of central sulcus interruptions. What could happen in case of very active expansion of the bridging gyrus is the apparition of a T-shape configuration at the end of the lower central sulcus root, definitively forbidding the connection with the upper root. A reversed T-shape could also appear at the bottom of the upper sulcal root. It seems to be the configuration observed for the two hemispheres of Fig. 2.

Interrupted sulci provide the opportunity to explore further the links between the bridging gyrus and the hand sensorimotor areas. What about configurations where this gyrus reaches the surface of the brain? If the bridging gyrus is really created by the growth of the hand sensorimotor area, the link should be preserved even in extreme configurations. To our knowledge, the literature includes only one case of interrupted central sulcus with fMRI-based sensorimotor mapping, but the results reported in 2D are difficult to interpret (Alkadhi and Kollias 2004). Therefore, we decided to look for interrupted central sulci in the outstanding Human Connectome Project dataset (Van Essen et al. 2012, 2013; Glasser et al. 2013). Subjects were chosen by the HCP consortium to represent healthy adults beyond the age of major neurodevelopmental changes and before the onset of neurodegenerative changes. All subjects provided written informed consent on forms approved by the Institutional Review Board of Washington University. We used the 820 subjects from the HCP-S1200 release labelled as Caucasian and already processed for a heritability study (Le Guen et al. 2018b). This set of 820 subjects raised the hope to fish about 8 interrupted central sulci. Furthermore, the HCP rich dataset provided us with the opportunity to validate the identification of interrupted central sulci using simple fMRI contrasts delivered by the consortium, mapping the hand, the foot and the tongue sensorimotor systems (Woolrich et al. 2001; Jenkinson et al. 2002; Feinberg et al. 2010; Moeller et al. 2010; Barch et al. 2013). Participants were presented with visual cues that ask them to tap their left or right fingers, squeeze their left or right toes, or move their tongue.

Since the central sulcus is usually not interrupted and relatively easy to identify, the study of the PPFM has often been performed using profiles of depth along the bottom line of the sulcus (Cykowski et al. 2008; Hopkins et al. 2010; Coulon et al. 2011; McKay et al. 2013; Hopkins et al. 2014; Schweizer et al. 2019). The rare cases of central sulcus interruption require a different strategy, all the more that the central sulcus automatic recognition may be problematic for such configurations. We leveraged a new approach developed to study asymmetry and heritability of the main plis de passage (Le Guen et al. 2018b).

**Fig. 4:**
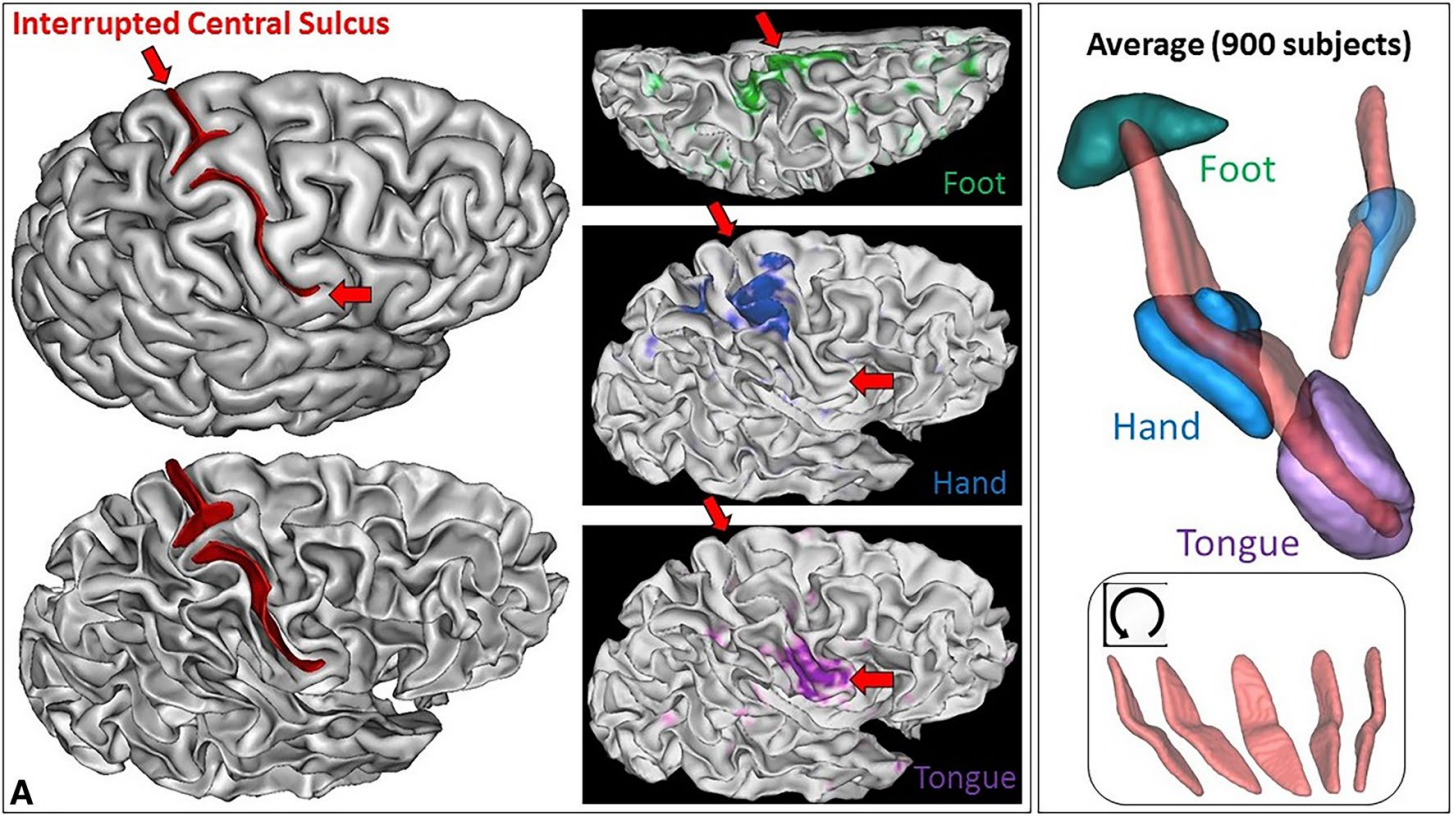
Left: one of the nine hemispheres of the HCP dataset with an interrupted central sulcus (BrainVISA viewer in the left, Connectome WB viewer in the middle). The foot, hand and tongue sensorimotor contrasts are used to validate the central sulcus identification (reported from BrainVISA viewer with the red arrows). Right: Average of the three sensorimotor fMRI contrasts computed from about 900 subjects after central sulcus alignment

**Fig. 5:**
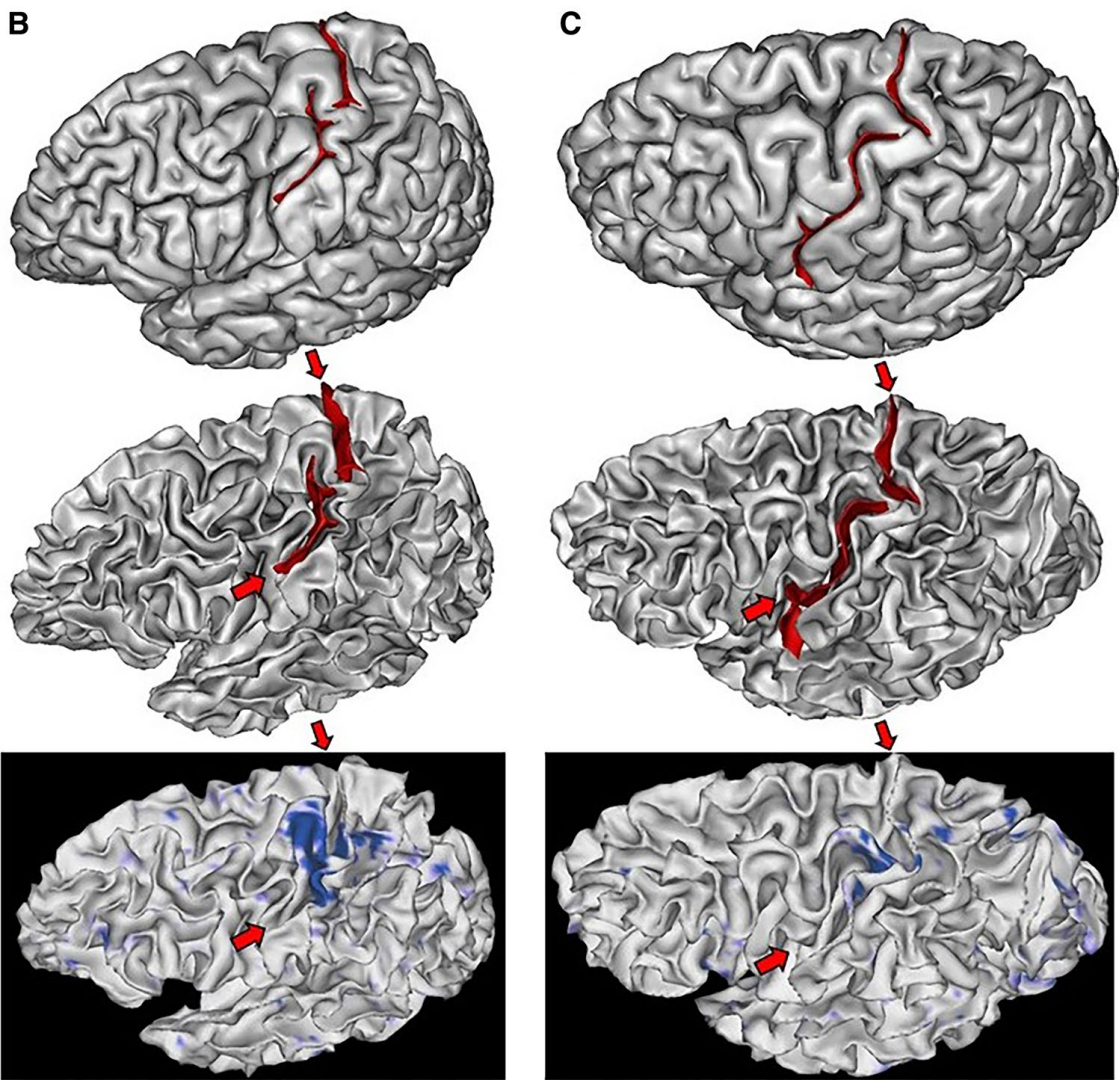
The two left hemispheres of the HCP dataset with an interrupted central sulcus. The two first lines are provided by Anatomist, the viewer of BrainVISA. The bottom line is provided by the Connectome Workbench viewer for the hand sensorimotor contrast

**Fig. 6:**
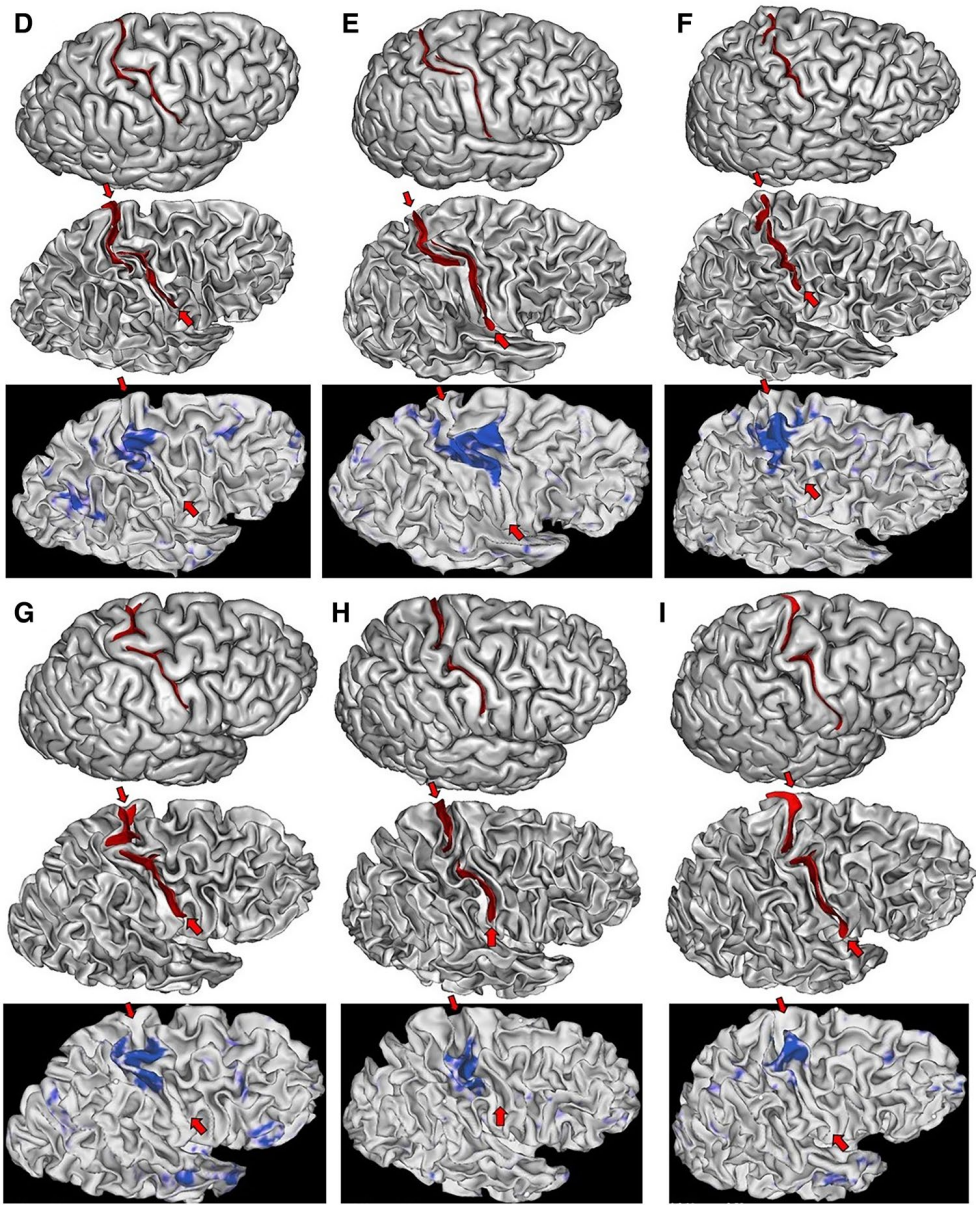
Six of the seven right hemispheres of the HCP dataset with an interrupted central sulcus. The two first lines are provided by Anatomist, the viewer of BrainVISA. The bottom line is provided by the Connectome Workbench viewer for the hand sensorimotor contrast

The sulcal pit map was detected first in each brain of the HCP dataset (Auzias et al. 2015) using the Cortical Surface toolbox of BrainVISA (http://brainvisa.info) applied to the meshes of the cortical surface computed with Freesurfer from the T1-weighted MRI (Fischl 2012; Le Guen et al. 2018b). Depth profiles were computed between the two main sulcal pits of the central sulcus (Le Guen et al. 2018b). Finally, the PPFM was detected as a significant depth minimum. Hence, rather than inspecting visually the 1640 hemispheres, we selected only the hemispheres with a candidate PPFM less than 5 mm deep, which corresponded to about 1% of the dataset. Each T1-weighted MRI was also processed using the Morphologist toolbox of BrainVISA, in order to extract the pial surface, the white matter surface, and a negative mold of the central sulcus used for visualization (Fischer et al. 2012). Half of the selected hemispheres were discarded because the central sulcus was clearly not interrupted. The candidate PPFM was an artefact probably resulting from a misalignment with Freesurfer template creating a spurious interruption. Note that only 0.5% of the hemispheres were clearly misaligned at the level of the central sulcus, which is without consequences in most neuroimaging studies. We were left with nine hemispheres with a complete interruption of the detected central sulcus. To validate the identification of the two pieces of the central sulcus, we used the connectome Workbench viewer of the HCP project (Marcus et al. 2011) to confirm that the foot, hand and tongue sensorimotor activations were located along the central sulcus (see Fig. 4), which happened for the nine hemispheres. For this visualization, we used the fMRI individual contrasts provided by the HCP consortium for a decimated version of the cortical surface mesh. To clarify the content of these three contrasts, we also computed an average across the 900 first subjects delivered by the HCP consortium (second release). For this purpose, we first aligned all the central sulci using the ICP algorithm (Sun et al. 2016, 2019). Then we projected the vertices of the cortical surfaces of all the subjects in the same space to compute an average (see Fig. 4). For the nine hemispheres, we could observe that the individual hand sensorimotor contrast in the central sulcus region was very similar to the average contrast of Fig. 4. Furthermore, the hand areas were clearly located in the bridging gyrus and extended downward in the postcentral gyrus like in the average (see Figs. 5, 6).

While the shape of the average central sulcus of Fig. 4 is very smooth, the association between the precentral motor side of the activation with the hand knob is clear. It was shown before using a manifold-based shape analysis that this association holds whatever the position of the knob along the central sulcus, which can vary from the middle of the sulcus to the upper two-thirds of the sulcus (Sun et al. 2016). The sensory side of the activation is much more elongated, covering the middle third of the postcentral gyrus, reaching much lower than the motor side. It should be noted that in the standard buried PPFM configuration, the gyrus buried in the postcentral wall is usually located lower than the gyrus buried in the precentral wall. Without fMRI tagging of these buried gyri, however, this observation is questionable, because other interdigitations can be observed in the central sulcus (see Fig. 1). The visualization of the average central sulcus from inside the brain (under the sulcus) highlights its twofold geometry. The hand sensorimotor activation seems located exactly where the two parts of the sulcus merge, which is supposed to be the localization of the deepest part of the PPFM.

We propose our visualizations of the nine hemispheres in Figs. 4, 5, 6, in order to allow observations of the folding pattern surrounding the central sulcus. It is the first time that several brains with this rare configuration are gathered. It is interesting to explore to which extent this configuration is a proxy for more global specificities. Some of the hemispheres present the “T-shape stop signal” observed in Fig. 2, but it is not systematic. It seems that some configurations correspond to cases where the two sulcal root were not able to meet. In such situations, the roots get much more elongated than usual (cases D & E). We found two interruptions in the left hemisphere versus seven in the right hemisphere, but this unbalanced result has to be reproduced.

The clear relationship between the PPFM and a regional feature of the cortical functional organization calls for a systematic exploration of the potential meaning of the other plis de passage. Interesting candidates are the plis de passage interrupting the Superior Temporal Sulcus, which are more developed in the left hemisphere, hinting at a link with the language system (Ochiai et al. 2004; Glasel et al. 2011; Segal and Petrides 2012; Leroy et al. 2015; Le Guen et al. 2018b). Another language-related area of interest is Broca’s area, where a submerged gyral passage can often be found separating the sulcus diagonalis from the neighboring sulcus (Sprung-Much and Petrides 2018). A number of plis de passage impacting the geometry of the precentral and the postcentral gyri would also be interesting targets (Germann et al. 2005; Amiez et al. 2006; Zlatkina and Petrides 2010; Zlatkina et al. 2016).

## Conclusion

The plis de passage appear in the earliest stages of development. Therefore, they should be regarded as a deep-seated character of the cortical organization that could help us to decipher the mechanisms driving the cortical folding process. The origin of the variability of their depth is unclear, but our speculations on the PPFM lead to a simple hypothesis: when the tangential expansion creating the bridging gyrus occurs early enough relative to the orthogonal expansion fathering the main sulcus, the pli de passage can resist burial, for instance thanks to T-shape local folding patterns. This chronological hypothesis may lead to think that the depth of the plis de passage is just an epiphenomenon. A recent result, however, has shown that a superficial pli de passage in the visual word form area has a positive impact on reading skills (Cachia et al. 2018).

Much more work is required to reveal the nature of the buried gyri. For instance, while the tension-based theory of D. Van Essen to explain the folding pattern has been set aside regarding primary sulci (Van Essen 1997; Llinares-Benadero and Borrell 2019), fiber-based constraints may be one of the drivers of secondary and tertiary folding. Hence, the potential relationships between plis de passage and U-fiber bundle organization is a very attractive research program (Mangin et al. 1998; Pron et al. 2018; Bodin et al. 2019). U-fiber organization must be impacted by the depth of plis de passage. Furthermore, the depth of a pli de passage may have an impact on the connectivity arising from the exuberant proliferation of axons occurring during development: the existence of a pli de “passage” reaching the brain surface may increase the amount of axons connecting the functional areas located on both side of the sulcus.

**Fig. 7:**
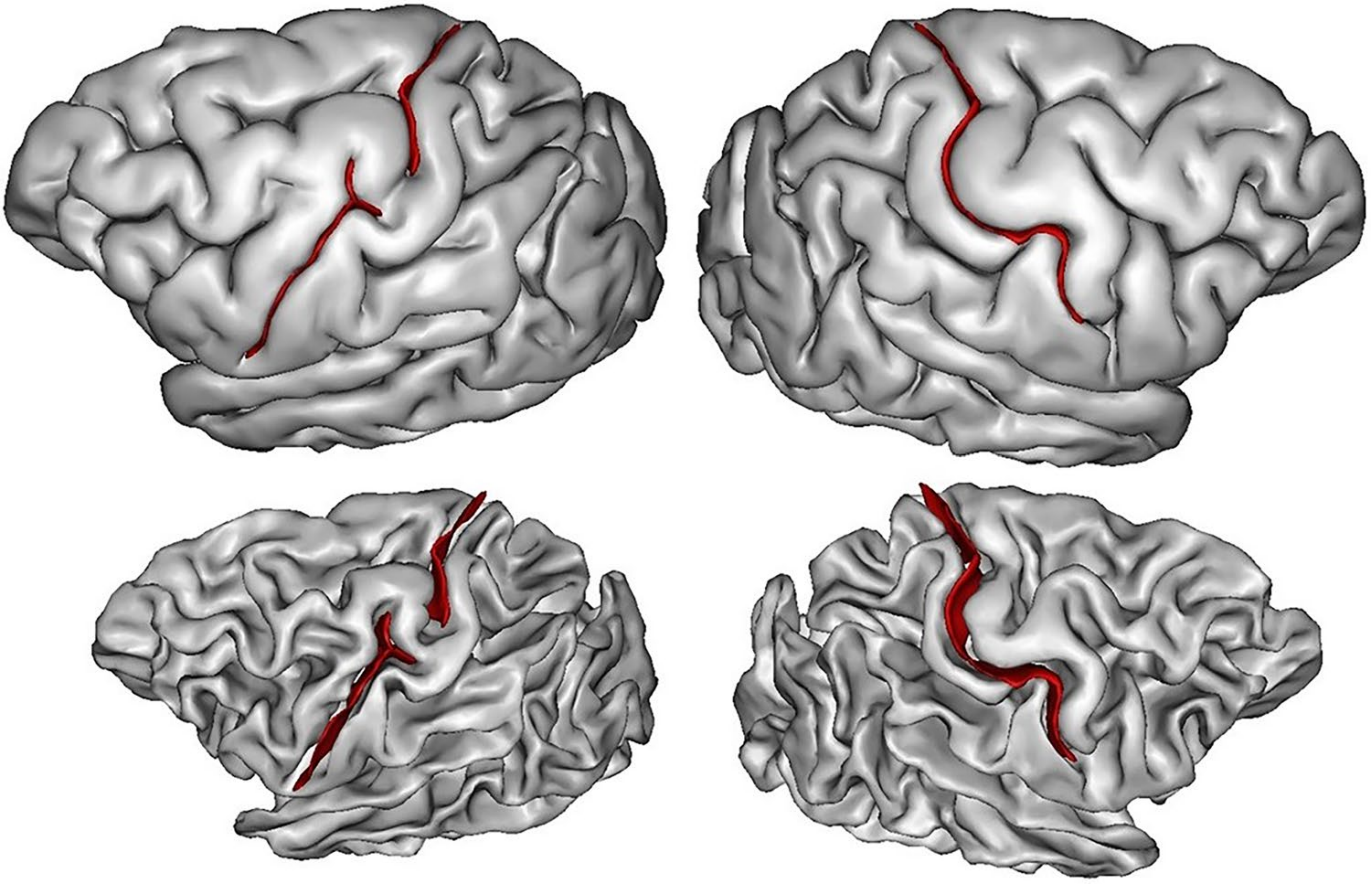
A chimp brain with central sulcus interruption in the left hemisphere

It would be also interesting to clarify whether some of the plis de passage are specific to humans. Cunningham mentioned in his seminal work that he did observe some of the plis de passage in chimp and orang brains (Cunningham 1890b). Browsing the chimp brain collection of W. Hopkins, we found a quasi-interrupted central sulcus (see Fig. 7). Hence, there may exist a marked tendency in the human brain towards the breaking up of the main sulci into two or more component parts by the formation of deep or superficial annectant gyri, but it may just be related to brain size. It would be interesting to test to which extent the pli de passage phenomenon is impacted by the folding allometries reported in the past (Toro et al. 2008; Germanaud et al. 2012, 2014).
